# The Changes of Twist1 Pathway in Pulmonary Microvascular Permeability in a Newborn Rat Model of Hyperoxia-Induced Acute Lung Injury

**DOI:** 10.3389/fped.2020.00190

**Published:** 2020-04-23

**Authors:** Ying Ruan, Wenbin Dong, Lan Kang, Xiaoping Lei, Rong Zhang, Fan Wang, Xiaodan Zhu

**Affiliations:** Department of Newborn Medicine, The Affiliated Hospital of Southwest Medical University, Luzhou, China

**Keywords:** bronchopulmonary dysplasia, hyperoxia, microvascular permeability, Twist1, Ang, Tie

## Abstract

**Background:** Bronchopulmonary dysplasia (BPD) is a chronic lung disease in preterm infants, which is characterized by alveolar and vascular dysplasia and increased vascular permeability. Hyperoxia is a critical factor in the pathogenesis of BPD, hyperoxia-induced acute lung injury (HALI) model has similar pathological manifestations as human BPD, therefore, may provide insight into the pathogenesis of human BPD. Studies have shown that Twist1 regulates pulmonary vascular permeability of LPS-induced lung injury through the Ang-Tie2 pathway. However, the effect of Twist1 pathway on vascular permeability in HALI has not been reported.

**Methods:** We randomly exposed newborn rats to the room air or hyperoxia for 14 days. Lung histopathology, immunofluorescence, vascular permeability, mRNA and protein expression was assessed on day 1,7,14.

**Results:** Our results verified that hyperoxia caused alveolar and vascular developmental disorders and increased pulmonary vascular permeability, which was consistent with previous findings. In hyperoxia-exposed rat lungs, the expressions of Twist1, Ang1, Tie1, Tie2, and pTie2 were significantly reduced, whereas the expression of Ang2 was significantly increased. Next, we observed a significant down-regulation of the Akt/Foxo1 pathway.

**Conclusion:** In HALI, the pulmonary microvascular permeability was increased, accompanied by changes in Twist1-Tie2 pathway which combined to Angs, and downregulation of Tie1 and Akt/Foxo1 pathway.

## Introduction

In the past few decades, although the survival rate of preterm infants has increased due to the milder oxygen therapy, prenatal steroids and postpartum pulmonary surfactant (PS), the incidence of the “new” BPD which is characterized by the alveolar simplification and disordered angiogenesis has increased year by year (1), which has become an essential factor affecting the survival quality of premature infants. Lung development includes two stages: alveolar development and pulmonary vascular development. At the same time as alveolar development, the pulmonary microvascular system expands synchronously with the alveolar septum, forming a blood-air barrier (2). There is evidence that the pulmonary microvascular network actively promotes the normal growth of alveoli in the process of lung development, and participates in the maintenance of alveoli structure after birth (3). Angiogenesis regulation plays a necessary role in the formation of the alveolar-capillary network. The deregulation of this process not only impairs the development of the pulmonary microvascular network but also leads to the formation of the immature alveoli, thus leading to lung pathologies such as BPD (4). The pathogenesis of BPD is complicated, the hyperoxia-induced acute lung injury (HALI) is a major contributor to the pathogenesis of BPD (5), which is characterized by increased pulmonary permeability and impairment of alveolar development (6). Therefore, the HALI model may provide insight into the pathogenesis of human BPD. In HALI, the pulmonary microvascular endothelium is damaged, leaving a bare capillary basement membrane area, resulting in increased permeability of the vascular endothelium (7). It has been further found that in the early stage of pulmonary edema of BPD, there is the destruction of the tight connection of the pulmonary vascular endothelium, and the abnormal expression of connexin Cx40 in the lung tissue, which may be related to the increase of the vascular permeability in the stage of pulmonary edema (8). Therefore, maintaining the integrity of the pulmonary microvascular barrier structure and reducing the microvascular permeability is the key to treatment of BPD. However, the specific mechanism of pulmonary vascular permeability changes in BPD is still unclear.

The bHLH transcription factor Twist1 was initially identified in Drosophila by the presence of twisted torsos in embryos that lacked the Twist1 gene (9). It regulates many biological processes such as the epithelial-mesenchymal transition in pulmonary fibrosis (10) and lung cancer (11), in which vascular permeability increases. These different functions of Twist1 are achieved through transcriptional regulation of its targets by recognizing a consensus E-box (CANNTG) motif on the promoter region of target genes to influence their transcription (12) such as tyrosine kinase receptor 2 (Tie2). Twist1 controls angiogenesis and endothelial cell (EC) sprouting through the angiopoietin (Ang)- Tie2 pathway, the disorder of this process mediates the pathological angiogenesis and collagen deposition in bleomycin-induced pulmonary fibrosis model in mice (13). It has also been found that the Twist1-Tie2 pathway is significant to control pulmonary vascular permeability. Twist1 knockdown disrupted cell junction integrity and increased vascular permeability by inhibiting Tie2 expression *in vivo* and *vitro* under physiological conditions (14). Although the Twist1-Tie2 signaling is evident in lung diseases, the role of this signaling in the changes of pulmonary microvascular permeability in HALI has been less explored. Therefore, we hypothesized that the Twist1-Tie2 signaling might participate in the changes of pulmonary vascular permeability in HALI. Given that the Ang-Tie-Akt/Foxo1 pathway plays an important role in the regulation of angiogenesis (15–18), in this research, we further detected whether the Akt-Foxo1 pathway was abnormal in HALI.

In the current study, we showed that the lungs of hyperoxia-exposed rats displayed histological changes consistent with BPD and increased pulmonary vascular permeability. In the lungs, the expressions of Twist1, Ang1, Tie1, Tie2, and pTie2 were significantly reduced, whereas the expression of Ang2 was significantly increased. Next, we observed a significant downregulation of the Akt/Foxo1 pathway. These findings suggest that in HALI, the pulmonary microvascular permeability was increased, accompanied by changes in Twist1-Tie pathway which combined to Angs, and downregulation of Tie1 and Akt/Foxo1 pathway, which may play a vital role in microvascular permeability of HALI.

## Materials and Methods

### Animal Model

A total of 24 time-dated, pregnant SD rats were purchased from the experimental animal center of the Southwest Medical University (Sichuan, China). All animal procedures were reviewed and approved by the Laboratory Animal Ethics Committee of the Southwest Medical University and conducted according to the AAALAC and the IACUC guidelines [License number of the experimental animal: SYXK (Sichuan) 2018-065]. All animals were housed at the experimental animal center of the Southwest Medical University and were permitted access to food and water ad libitum at a temperature range of 20–23°C under a 12:12 h light-dark cycle.

A total of 114 full-term newborn rats were randomly and equally assigned to hyperoxia group and air group within 12 h after birth according to the random number table method. The HALI model was established by high oxygen treatment (8, 19). The hyperoxia group was placed in a closed oxygen tank, and 2L/min of oxygen was continuously introduced to maintain the oxygen concentration at 80~90%. Sodium lime was used to absorb excessive carbon dioxide. The air group was placed in the same room air (21% oxygen). The nursing mothers were switched every 24 h between the room air and hyperoxia-exposed litters to avoid oxygen toxicity and to eliminate maternal effects. Chamber was opened for 30 min/day for cage cleaning. Nineteen pups were obtained randomly in each group and sacrificed by intraperitoneal sodium pentobarbital injection at the end of 1, 7, 14 days of exposure.

### Animal Growth

The survival number, mental state, reaction, feeding status, growth status, and the bodyweight of the newborn rats were recorded every day. At 1, 7, and 14 days of hyperoxia, sixteen newborn rats were randomly taken from each group, and the bodyweight at the time of sampling was recorded. Weight gain is expressed as a weight gain rate, i.e., body weight growth rate = (body weight at sampling—birth weight)/birth weight.

### Sample Collection and Preparation

The lungs were harvested from each group at different time points. The left lung tissues of each group were inflated through the trachea with 4% paraformaldehyde and fixed overnight in the same solution. The fixed tissues were dehydrated and cleared, and five lung tissues were paraffin-embedded for hematoxylin and eosin staining (HE), three lungs were embedded in OCT for immunofluorescence (IF). The lung and body weights of the newborn rats from each group (without formalin fixation) were measured for lung weight /body weight (LW/BW) ratio calculations as an index of lung injury. The remaining lung tissues were immediately frozen at −80°C for reverse transcription-quantitative polymerase chain reaction (RT-qPCR) analysis and immunoblot analysis.

### Histopathology and Immunofluorescence Analysis of Lungs

For HE, the paraffin-embedded lung tissues were cut into 4-μm in thickness sections, which were stained with HE for histological analyses of lung injury. From each section, 5–10 random areas were examined at × 20 magnification with an optical microscope. The level of alveolar development was assessed using the mean linear intercept (MLI) methods (20). Ten fields of view were taken at random with the 20 objective on each HE-stained section. Draw a cross line in the center of the collected picture and count the total length of the cross line and the total number of alveolar septa that intersect with the cross line. The MLI calculation method for each field of view: the total length of the cross line divided by the total number of alveolar septa. Finally, the MLI of each section was calculated as the average of the MLI of the 10 fields of view. Large trachea and blood vessels are not included in the calculated field of view.

For IF, the OCT-embedded lung tissues were frozen at −20°C. The frozen tissues were cut into 10-μm sections with a cryostat. The sections were washed with PBST 3 times for 5 min to eliminate OCT, and then incubated in 0.5% TritonX-100 (Amresoo, Washington, USA) for 20 min. The sections were then washed 3 times for 5 min with PBST, blocked with goat serum for 1 h, and incubated with primary antibody. The sections were washed 3 times for 5 min with PBST, and then incubated with fluorescent secondary antibody for 1 h. Finally, the sections were washed 3 times for 5 min with PBST, and then added anti-fluorescence quenching sealing liquid (including DAPI) for sealing. CD31 was detected withing an anti-CD31 primary antibody (1:100, Santa Cruz Biotechnology, CA, USA) and a Rhodamine (TRITC)-conjugated goat anti-rat IgG(H+L) (1:100, Proteintech Group, Inc, USA) secondary antibody. Twist1 was detected withing an anti-Twist1 primary antibody (1:50, Abcam, Cambridge, UK) and a Fluorescein (FITC)-conjugated affinipure goat anti-mouse IgG(H+L) (1:100, Proteintech Group, Inc, USA) secondary antibody. Tie1 was detected withing an anti-Tie1 primary antibody (1:100, Merckmillipore, Germany) and a Coralite488-conjugated affinipure goat anti-rabbit IgG(H+L) (1:250, Proteintech Group, Inc, USA) secondary antibody. Tie2 was detected withing an anti-Tie2 primary antibody (1:100, Merckmillipore, Germany) and a Fluorescein (FITC)-conjugated affinipure goat anti-mouse IgG(H+L) (1:100, Proteintech Group, Inc, USA) secondary antibody. From each section, at least three random areas were examined at × 40 magnification with a fluorescence microscope.

### Pulmonary Permeability Assay

Evans blue dye (EBD) can be tightly bound to albumin, and is a sensitive marker of early pulmonary edema and can be used to reflect vascular permeability (21, 22). Lung permeability was measured by LW/BW and by the Evans blue dye (EBD) (Biotopped, Beijing, China) leakage method (23). The lung tissues were homogenated and mixed with formamide (Amresoo, Washington, USA) (1 ml/100 mg tissue), incubated in a 60°C water bath for 24 h, then centrifuged in 5,000 g at room temperature for 30 min, and the absorbance of the supernatant was measured at 620 nm. A standard curve was made according to a series of dilutions of EBD. The concentration of EBD in each group was measured by the standard curve to evaluate the pulmonary vascular endothelial permeability.

### Reverse Transcription-Quantitative Polymerase Chain Reaction (RT-qPCR)

The mRNA levels of Twist1, Ang1, Ang2, Tie1, Tei2 were measured by RT-qPCR. The total RNA of each group was extracted from the snap-frozen lung tissues using TRIzol reagent (TIANGEN, Beijing, China) and frozen at −80°C. A total of 1,500 ng RNA from each sample was reverse transcribed to produced cDNA using ReverTra Ace qPCR RT Master Mix (TOYOBO, Japan). The cDNA was then amplified using THUNDERBIRD SYBR qPCR Mix (TOYOBO, Japan). Real-time PCR was conducted using qTOWER 2.0 Real-Time PCR System (Analytik Jena AG, Germany) with Gapdh as an internal control. The amplification reaction was performed as follows: 40 cycles of 95°C for 30 s, 95°C for 5 s, and 60°C for 10 s; and 72°C for 30 s. The relative quantification of mRNA expression for Twist1, Ang1, Ang2, Tie1, Tie2 was conducted using the 2^ΔΔCt^ method following normalization with Gapdh. The specific primers were designed as follows: Twist1, CCGGAGACCTAGATGTCATTGT (forward) and CTGGGAATCTCTGTCCACCG (reverse); Ang1, GGAGTCCAGAAAACGGAGGG (forward) and TTCTCAAGTTTTTGCAGCCAC (reverse); Ang2, CATGATGTCATCGCCCGACT (forward) and TCCATGTCACAGTAGGCCTTG (reverse); Tie1, AGAGACCACGCTGGGTAATG (forward) and CTTCACCCGATCCTGACTGG (reverse); Tie2, AAGAGCGAGTAGACCATGCG (forward) and ACTAGTCCATAAAGGAGCAAGC (reverse); Gapdh, GCAAGTTCAACGGCACAG (forward) and GCCAGTAGACTCCACGACAT (reverse).

### Western Bolt

Lung lysate extracts were made, and protein concentration determined by the BCA method. Proteins were separated by SDS-PAGE (10%) and transferred to PVDF membranes followed by 1 h blocking at room temperature (RT) in 7% non-fat milk of PBS with 0.07% Tween-20, and then incubated with anti-Twist1 antibody (1:500, Abcam, Cambridge, UK), anti-Angiopoietin 1 antibody (1:1000, Boster Biological Technology Co., Ltd., China), anti-Angiopoietin 2 (1:1000, Proteintech Group, Inc, USA), Tie1 antibody (1:1000, Thermo Fisher Scientific, Waltham, MA), anti-Tie2 antibody (1:1000, Merckmillipore, Germany), anti-phospho-Tie2 (Ser1119) antibody (1:500, Merckmillipore, Germany), phospho-Akt (Ser473) and Akt antibodies (1:1000, Cell Signaling Technology, Danvers, MA), phospho-FoxO1 (Ser256) and FoxO1 (C29H4) Rabbit mAb Antibodies (1:1000, Cell Signaling Technology, Danvers, MA), Gapdh Mouse Monoclonal Antibody (1:14000, Proteintech Group, Inc, USA) at 4°C, overnight. The following day membranes were washed three times with PBST (Phosphate buffer saline with 0.07% Tween 20), incubated with HRP conjugated secondary antibody [HRP-labeled Goat Anti-Rabbit IgG(H+L) or HRP-labeled Goat Anti-Mouse IgG(H+L); both Beyotime, Shanghai, China] for 1 h at RT, washing and then were measured by ECL chemiluminescence method. The gel analysis imaging system was used for scanning and analysis. The relative protein expression levels of target proteins were normalized to Gapdh.

### Statistical Analyses

The experimental data were processed by Graphpad prism8.0 software and Image J software and analyzed by SPSS 21.0 statistical software. The values were expressed as mean ± standard deviation. Independent sample *t*-test was used for the comparison between the two groups when normality and homogeneity of variance assumptions were satisfied; otherwise, the non-parametric test Mann-Whitney was used for analysis. In addition, the Kaplan-Meier method was used for survival analysis of each group, and the rank sum test was used for comparison of survival curves between the two groups. *P* < 0.05 was considered statistically significant.

## Results

### Hyperoxia Exposure Affected the General Health Conditions of the Newborn Rats

**Figure 1:**
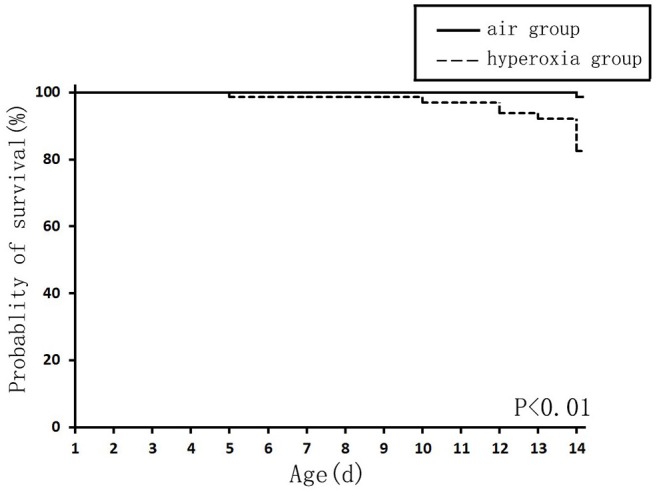
The survival curve of newborn rats in the hyperoxia and air group. The survival rate of the hyperoxia group was significantly lower than that of the air group, *P* < 0.01.

**Figure 2:**
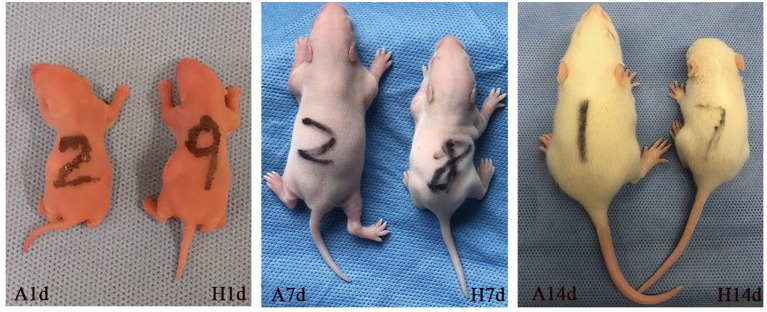
The general health conditions of the newborn rats at 1, 7, and 14 d after oxygen exposure. There was no difference between the two groups at 1 d. Newborn rats in the hyperoxia group (H) showed obvious poor development, small body size, inadequate mental response at 7 d, and the difference between the two groups was more evident at 14 d of oxygen exposure. In contrast, the air group (A) has good growth and development.

**Figure 3:**
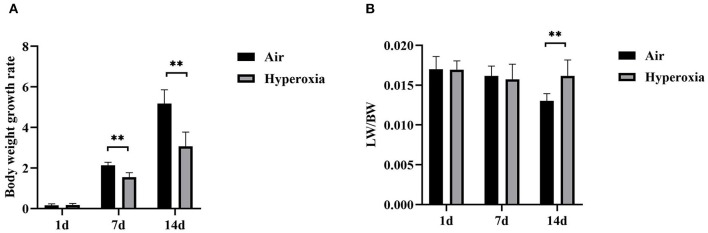
The body weight growth rate and lung/body weight ratios of the air and hyperoxia groups. **(A)** The body weight growth rate of the air and hyperoxia groups at different time points. **(B)** The lung/body weight ratios (LW/BW) of the air and hyperoxia groups at different time points. (**P < 0.001).

The survival rate of newborn rats in the hyperoxia group was significantly lower than that in the air group. The survival rates at 1, 7, and 14 d were 100, 98.56, 82.94%, respectively. With the extension of the hyperoxia time, the newborn rats' mortality gradually increased, and the survival rate gradually decreased. However, no death occurred in the air group at 1 and 7 d, and the survival rate at 14 d was 98.72% (Figure 1). We observed the mental state, activity, and hair luster of the newborn rats. With the prolongation of oxygen exposure time, the newborn rats in the hyperoxia group gradually showed weak milk seeking ability, decreased activity, slow response, dull hair, and thin body. Furthermore, from the 7 d after oxygen exposure, the neurological symptoms such as head fibrillation and gait instability began to appear, while the growth and development of the newborn rats in the air group were good, and the above abnormal symptoms did not appear (Figure 2). There was no difference in body weight of the newborn rats in both groups at 1 d. The body weight in both groups increased at 7 and 14 d after oxygen exposure compared with 1 d. However, the growth rate of the newborn rats in the hyperoxia group was significantly slower than that in the air group, and the difference between the two groups gradually increased with the extension of the experimental time (Figure 3A).

### Hyperoxia Could Cause Alveolar Simplification and Lung Injury in Newborn Rats

**Figure 4:**
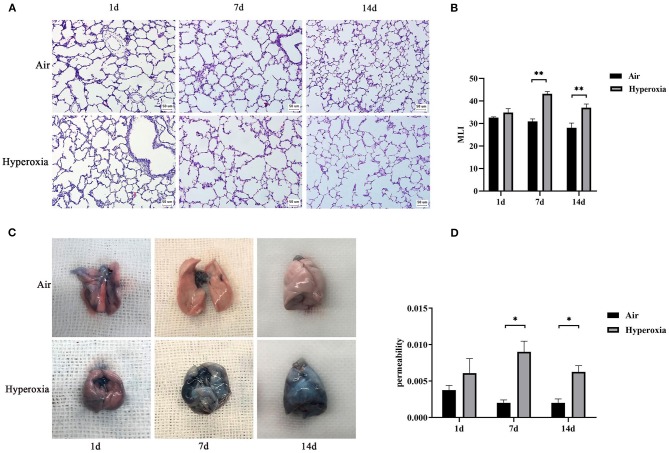
Induction of alveolar simplification and lung injury in newborn rats following hyperoxia. **(A)** The pathological changes of lung tissue were observed by hematoxylin-eosin staining in the air or hyperoxia group. Magnification, × 20. Hyperoxia group at 7 and 14 d showed increased lung injury with disordered lung structure, increased alveolar simplification, and alveolar size. **(B)** The effects of hyperoxia on MLI, which is an index of lung injury, were determined in newborn rats(*n* = 3–5/group). **(C)** Comparison of Evans blue staining in the lungs of the two groups at different time points. **(D)** Graph showing vascular permeability in the lungs of the two groups at different time points. Vascular permeability was detected by Evans blue dye leakage in the lungs, and extracted dye contents were quantified by measuring at 620 nm. *P < 0.05, **P < 0.001.

To determine the role of hyperoxia in lung injury, we analyzed the histological examination of the newborn rats' lungs exposed to air or hyperoxia after birth. It could be seen by the naked eye that the lung tissue of the newborn rats in the air group was pink and elastic, while that of the hyperoxia group was white and less elastic than that of the air group. Under the light microscope, the lung tissue structure of the newborn rats in the air group was gradually completed at 7 d. The alveoli were well-developed and uniform in size. At 14 d, the structure of the alveoli was clear and regular in shape, and the alveolar cavity was small. The alveolar septum was thick, and there was no inflammatory exudation in the alveolar space. In the hyperoxia group, the alveolar space increased, and the alveoli wall became thinner at 7 d. With the extension of oxygen exposure time to 14 d, the lung tissue structure was disordered, and the alveoli structure was simplified and uneven in size. Besides, the alveolar space was significantly enlarged, some of the alveoli fused, the number of alveoli decreased, and inflammatory cells and red blood cells exuded from the alveoli (Figure 4A). Upon morphometric analysis, a significant increase in the mean linear intercept (MLI) was noted in newborn rats exposed to hyperoxia (Figure 4B). The Lung weight /Bodyweight (LW/BW) ratio is an index of increased cellularity and pulmonary edema; therefore, it can be used to reflect the extent of lung injury. The LW/BW ratios of the newborn rats from the 14 d of the hyperoxia group indicated a significant increase compared with the air group suggesting an increase in lung injury of these animals (Figure 3B). The above results indicated that hyperoxia induced lung injury.

### Hyperoxia Increased Vascular Permeability in the Newborn Rats' Lung

To further determine whether hyperoxia regulates pulmonary vascular permeability, we measured vascular permeability in newborn rats' lungs by measuring the leakage of Evans blue dye (Figure 4C). The leakage of Evans blue dye into lung extravascular space was higher in the lungs of the newborn rats exposed to the hyperoxia compared to that in the air controls at the same time point suggesting that the permeability of pulmonary blood vessels to macromolecules such as Evans blue is gradually increased (Figure 4D). In addition, the ratio of LW/BW can also be used to reflect the pulmonary vascular permeability (24). As shown in Figure 3B, the LW/BW ratio of the newborn rats on the 14th day is significantly higher than that of the air group. The results above suggest that hyperoxia leads to the increase of pulmonary vascular permeability of the newborn rats.

### Hyperoxia Caused Reduced Expression of Twist1, Tie1, and Tie2 in CD31-Positive Cells

**Figure 5:**
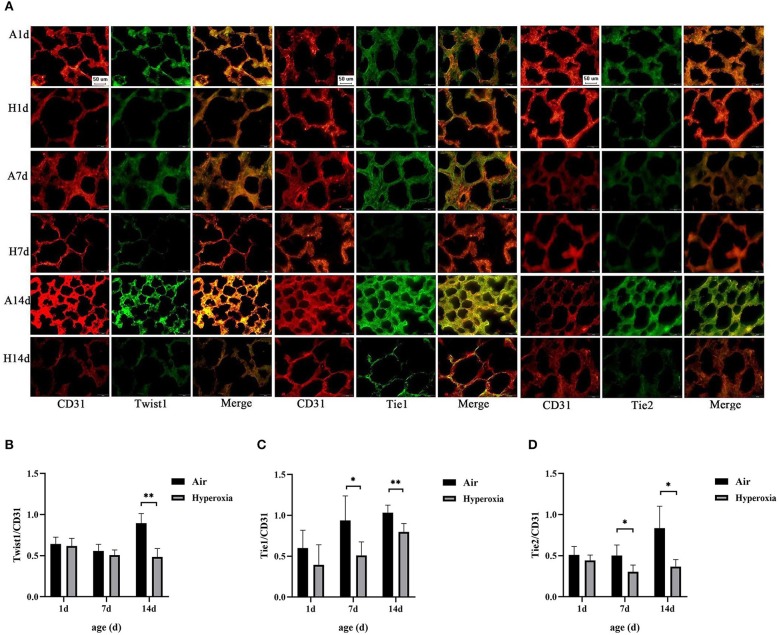
Hyperoxia caused reduced expression of Twist1, Tie1, and Tie2 in CD31-positive cells. **(A)** Immunofluorescence micrographs showed the expression and distribution of Twist1, Tie1, Tie2 in CD31-stained blood vessels of the lungs of the two groups at different time points (Magnification, ×40; bar, 50 μm). **(B)** The expression ratio of Twist1 in CD31 positive endothelial cells of the two groups at different time points (*n* = 3/group). **(C)** The expression ratio of Tie1 in CD31 positive endothelial cells of the two groups at different time points (*n* = 3/group). **(D)** The expression ratio of Tie2 in CD31 positive endothelial cells of the two groups at different time points (*n* = 3/group). *P < 0.05, **P < 0.001.

We examined the effects of hyperoxia exposure on the levels of Twist1 and Tie receptors in the lung tissues of the newborn rats by IF. IF detection in tissue sections showed that the lungs exposed to high oxygen failed to form septation, resulting in fewer and larger alveoli with less developed vascular structures (CD31-staining vessels), similar to the lungs of premature infants with BPD. The results also showed that the Twist1, Tie1, and Tie2 were specifically expressed in vessel ECs (CD31 positive cells). The Twist1, Tie1, and Tie2 expression were lower in CD31-positive endothelial cells of the hyperoxia group compared to those of the age-matched air controls (Figures 5A–D).

### Hyperoxia Down-Regulated Pulmonary Twist1, Tie1, Tie2, and Ang1 mRNAs, While Up-Regulated Ang2 mRNA

**Figure 6:**
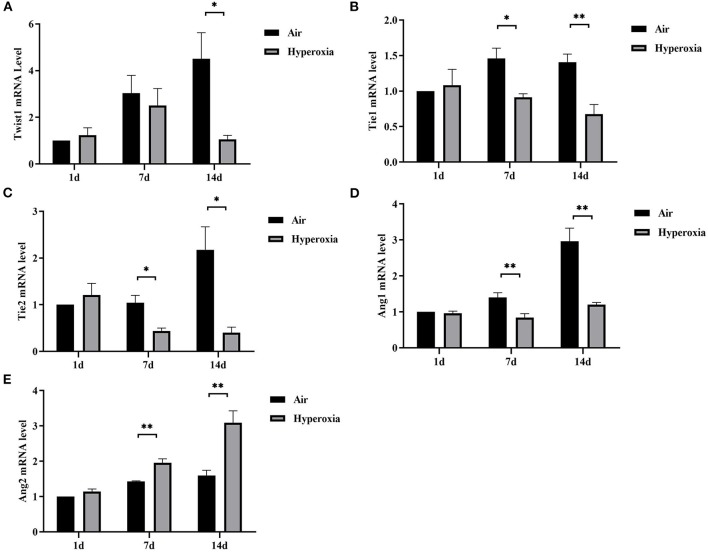
Hyperoxia down-regulated pulmonary Twist1, Tie1, Tie2, and Ang1 mRNAs, while up-regulated Ang2 mRNA. Lungs from all different groups were excised, total RNA was isolated, and Twist1 **(A)**, Tie1 **(B)**, Tie2 **(C)**, Ang1 **(D)**, and Ang2 **(E)** mRNA levels were determined by real time-PCR following cDNA synthesis, as described in the Materials and Methods section (*n* = 6/group). *P < 0.05, **P < 0.01.

Given that hyperoxia could cause pulmonary microvascular endothelial damage, resulting in increased vascular permeability. Therefore, we hypothesized that the Twist1 target gene Tie2 in conjugation with its ligands (Ang1/2) might play a crucial role in hyperoxia-induced pulmonary vascular permeability. To further investigate the mechanism of changes in pulmonary vascular permeability during hyperoxia, we evaluated the pulmonary mRNA levels of Twist1, Tie1, Tie2, Ang1, and Ang2. No significant decrease in the pulmonary Twist1 mRNA level was noted at 1 or 7 d in the hyperoxia group compared with the air controls. However, the Twist1 mRNA level in the hyperoxia group was significantly lower than that in the air group at 14 d. From the 7 d, hyperoxia reduced Tie1, Tie2, Ang1 mRNA significantly compared with the air controls, while induced Ang2 mRNA significantly (Figures 6A–E).

### Hyperoxia Reduced Pulmonary Twist1, Tie1, Tie2 and Ang1 Protein Levels, While Up-Regulated Ang2 Protein Level, and Inhibited the Expression of Proteins in Akt/Foxo1 Pathway

**Figure 7:**
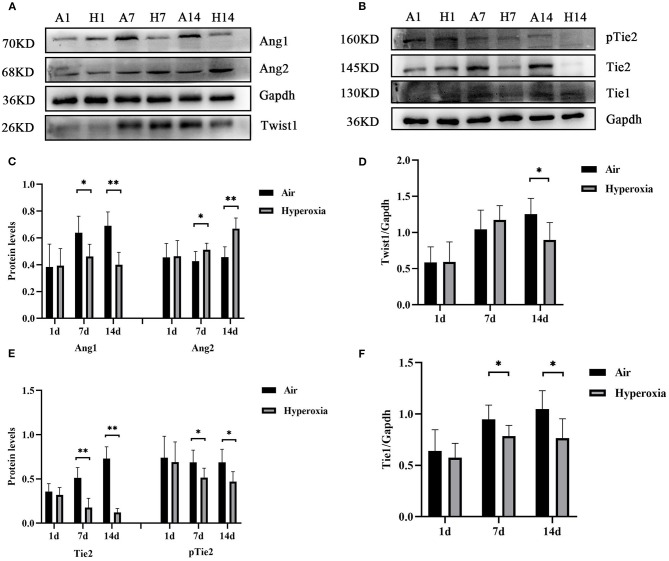
Hyperoxia reduced pulmonary Twist1, Tie1, Tie2, and Ang1 protein levels, while up-regulated Ang2 protein level. The lung homogenates prepared from different groups of newborn rats were subjected to western blots analysis. Western blots showed the expression of Twist1 **(A,D)**, Ang1/2 **(A,C)**, Tie1 **(B,F)**, pTie2/Tie2 **(B,E)** in the lungs. The densitometric intensities of these proteins normalized to Gapdh were quantified and shown separately (*n* = 8–14/group). *P < 0.05, **P < 0.01.

**Figure 8:**
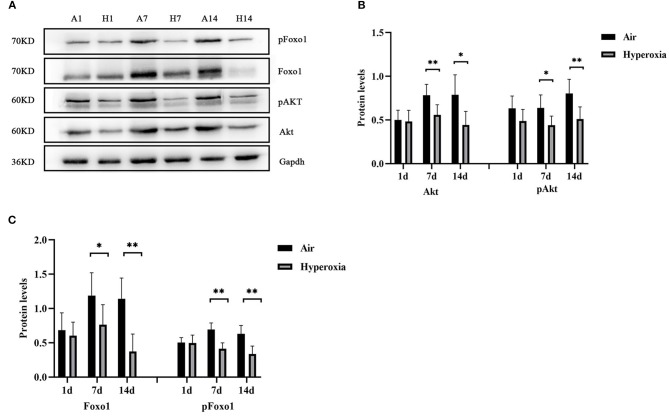
Hyperoxia inhibited the expression of proteins in AKT/Foxo1 pathway. Western blots showed the expression of pAkt/Akt **(A,B)**, pFoxo1/Foxo1 **(A,C)** in the lungs. The densitometric intensities of these proteins normalized to Gapdh were quantified and shown separately (*n* = 8–14/group). *P < 0.05, **P < 0.01.

To further determine the mechanism of Twist1 and its downstream signaling pathways in hyperoxia-induced pulmonary vascular permeability, we evaluated the protein expression in this signaling pathway in the air and hyperoxia groups at different time points by western blot. As shown in Figure 7, there was no significant difference in the expression of Twist1 at 1 and 7 d of the hyperoxia group compared with the age-matched air group. With the extension of oxygen exposure time, Twist1 decreased significantly at 14 d of hyperoxia compared with the air group at the same time point (Figures 7A,D). Similarly, at 1 d of hyperoxia, the protein levels of Ang1, Ang2, Tie1, pTie2, and Tie2 were not significantly different from those of the air group. However, with the prolongation of oxygen exposure time, the expression of Ang1, Tie1, ptie2, and Tie2 gradually decreased from the 7 d of hyperoxia, which was significantly different from that of the age-matched air group (Figures 7A–C,E,F). Conversely, the expression of Ang2 was significantly higher than that of the air group at 7d of hyperoxia and continued to increase at 14 d of hyperoxia (Figures 7A,C). Given that the Tie2 can regulate the activity of Akt and its downstream target protein Foxo1, we also detected the change of Akt/Foxo1 pathway protein in hyperoxia group. Similarly, with the prolongation of oxygen exposure time, the expression of pAkt, Akt, pFoxo1, and Foxo1 proteins decreased significantly from 7 d of hyperoxia, which was significantly different from that of the air group at the same time point (Figures 8A–C).

## Discussion

BPD is a chronic lung disease in infancy that is associated with significant mortality and long-term morbidity. Previous studies on BPD mainly focused on the mechanism of alveolar dysplasia. However, with the development of new BPD, pulmonary vascular development has gradually received attention, and tight regulation of vascular permeability is the key to maintain lung function. Previous studies have confirmed that the alveolar development process of rats has a similar chronology to humans, the lung development of term newborn rats is in the saccular stage, which is equivalent to that of human at 28 weeks of gestational age (25). Fourteen days after birth is the key period of alveolar formation, the pulmonary blood vessels have also entered a period of significant proliferation (26). It has been reported that the HALI model in which newborn rats were continuously exposed to 85–90% hyperoxia could simulate the BPD-like lung injury (8, 19, 27). Therefore, in this study, we exposed term newborn rats to 85–90% hyperoxia for 14 days to establish a HALI model. Our data showed a significantly reduced body weight in the hyperoxia group compared with the air group. The newborn rats' mortality gradually increased with the extension of the hyperoxia time. The main pathological changes of BPD are impaired development of alveolar and vascular. Hyperoxia induced decreased number and increased volume of alveolar, disordered lung structure, pulmonary fibrosis and pulmonary vascular dysplasia in newborn rats (27, 28). Our HE staining showed that the obvious structural disorder and simplification of alveolar structure in the lung tissues appeared in the hyperoxia group from the 7th day, and the MLI value was significantly higher than that in the air group, which further indicated that the number of alveoli decreased, reflecting the hypoplasia of lung. The above further proved that the HALI model was successfully established.

In addition, we found that the lung EBD extravasation and LW/BW value of the newborn rats increased after exposure to high oxygen, confirming that pulmonary vascular permeability was increased in HALI (6, 7). Our immunofluorescence results showed that hyperoxia reduced the expression of Twist1, Tie1, and Tie2 in CD31 positive blood vessels. In the lung tissue, CD31 is mainly expressed in pulmonary microvascular endothelial cells and is often used as a marker of vascular endothelial cells (29). With the increase of oxygen exposure time, the CD31 positive blood vessels in the lung tissue of the hyperoxia group were lower than those in the air group, suggesting that there might be pulmonary vascular development obstruction in HALI. Then we examined the changes in mRNA and protein levels of Twist1, Ang1, Ang2, Tie1, and Tie2 in lung tissues. The results showed that the levels of Twist1 mRNA and protein in the hyperoxia group were significantly lower than those in the air group at 14 d. At 7 and 14 d, the mRNA and protein levels of Ang1, Tie1, Tie2, and pTie2 in the hyperoxia group were significantly lower than those in the air group at the same time point. On the contrary, the levels of Ang2 mRNA and protein increased significantly at 7 and 14 d after hyperoxia exposure. In recent years, Twist1 has become a hot spot in the research of vascular-related diseases. It may control the formation of blood vessels by regulating the expression of many angiogenic factors and receptors in endothelial cells, such as platelet-derived growth factor B (PDGFB), vascular endothelial growth factor receptor 2 (VEGFR2), Tie2 (14, 30). In cancer and hyperoxia-induced retinopathy models, Twist1 mediated pathological angiogenesis by changing the expression of VEGF and VEGFR2 (30–32). Mammoto et al. (14) found that knockout of Twist1 *in vitro* cultured lung human microvascular endothelial (L-HMVE) cells and *in vivo* adult mouse lung could reduce Tie2 and its phosphorylation status, leading to increased pulmonary vascular permeability in physiological condition where Ang1 is dominant. However, in pathological condition such as endotoxin-induced lung injury, Ang2 was upregulated, knockout of Twist1 failed to increase the pulmonary vascular permeability. These findings demonstrated that the Twist1-Tie2 pathway combined with the Angs controlled the pulmonary vascular permeability in an environment-dependent manner. Angs and Tie receptors are widely expressed in lung tissues, and deregulation of this system contributes to the pathogenesis of various lung diseases such as acute lung injury (ALI) and BPD (33, 34). Ang1 and Ang2 compete with Tie2 receptors to participate in the regulation of endothelial barrier function. Ang1 is secreted by smooth muscle cells and mesenchymal cells around blood vessels (35). As an obligatory Tie2 agonist, Ang1 promotes the germination and branching of blood vessels and vascular stability and maturation, inhibits endothelial cell apoptosis by activating its specific receptor Tie2 (36). In physiological conditions, Ang1 is dominant compared to Ang2 and more likely to combine with Tie2, and induces the transfer of the Tie2 receptor to cell-cell contact or cell-matrix interface, to stabilize the integrity of endothelial cell connection (37, 38). Studies have shown that Ang1 could inhibit the permeability of paracellular cells induced by a variety of inflammatory cytokines and growth factors such as thrombin, histamine, and VEGF (39, 40). Therefore, Ang1 plays a vital role in maintaining the continuity and integrity of new blood vessels. In contrast, Ang2 only plays a weak agonist role for Tie2, it almost does not activate Tie2 in physiological condition, it is related to vascular instability and remodeling (41). Ang2 is expressed by ECs and is stored in the endocrine granules in ECs called Weibel Palade body, which can be rapidly secreted when stimulated by inflammatory factors (42). Ang2 promotes vascular leakage by cooperating with inflammatory cytokines. In pathological conditions where Ang2 is dominant, Ang2 combined with Tie2 to play an antagonistic role, making ECs respond quickly to local and systemic stimuli, and destroying the occurrence of connexins between ECs, resulting in increased vascular permeability (43, 44). In our experiment, the Ang1-Tie2 pathway was downregulated in the lungs of newborn rats exposed to hyperoxia, which was consistent with a previous study (34). However, Ang2 was upregulated, Twist1 and Tie2 was downregulated, while the pulmonary vascular permeability was still increased in HALI, this seemed to contradict the conclusion of the research by Mammoto T et al. The possible reasons may be that, in the hyperoxia group, the decrease of Ang1 expression could weaken the Ang1-Tie2 signal, which might lead to vascular development disorder and increase of vascular permeability. Besides, because Ang1 and Ang2 bind to Tie2 in a similar way (45, 46), the binding of Ang2 to Tie2 might lead to the inhibition of the signal transduction of Tie2 induced by Ang1 when the ratio of Ang2/Ang1 increased and make Ang2 play an antagonistic role of Tie2, thus making vascular instability. As a regulator of Tie2 expression, the reduced Twist1 might downregulate the Tie2 expression, which possibly reduce the antagonistic effect of Ang2 on Tie2, and decrease the damage of Ang2 on vascular permeability. Therefore, we speculated that in physiological conditions dominated by Ang1, Twist1 was essential to maintain the stability of pulmonary vessels induced by Ang1-Tie2 signal. In hyperoxia conditions, in which Ang2 was upregulated, the reduction of Twist1might play a compensatory protective role to resist the damage of hyperoxia on pulmonary vascular permeability. Maybe in the natural state without knocking down the Twist1, the decreased extent of Twist1 induced by hyperoxia is not enough to reduce Tie2 to reverse the increase of vascular permeability induced by Ang2-Tie2.

Our experimental data showed that Twist1 had significant difference between the two groups at 14 d, while Ang1, Ang2, Tie2 was significantly different at 7 and 14 d. In the research by Mammoto T et al. knocking down Twist1 did not alter the Ang1 and Ang2 expression, the levels of Ang1 and Ang2 just determined the effect of the Twist1-Tie2 pathway on vascular permeability. As a regulator of Tie2, the decrease of Twist1 and Tie2 was not synchronous, we analyzed that there were other factors that could regulate Tie2 expression in addition to Twist1, such as micro-RNA (miR)-34a (34). Ang1 and Tie2 were targets of miR-34a, they had conserved miR-34a seed sequence in its 3′UTR. MiR-34a could stimulate cell death and reduce the expression of target protein Ang1 and Tie2. In HALI, micro RNA (miR-34a) was significantly upregulated and Ang1/Tie2 expression was decreased. However, inhibition of miR-34a reduced cell death and enhanced the expression of Ang1/Tie2 in hyperoxia conditions. Therefore, in our experiment, we speculated that both micro RNA (miR-34a) and Twist1 might regulate the Tie2 expression with the prolongation of hyperoxia exposure time. It has not been found that Twist1 can regulate the expression of Tie1. The mechanism of the decline of Tie1 caused by hyperoxia is unclear. Some studies have demonstrated that Tie1 plays an indispensable role in angiopoietin signaling during vascular remodeling and ECs proliferation (47). After treatment with Ad-CAng1 or Ad-Ang2, the tracheal blood vessels of the mice enlarged significantly and endothelial cell proliferation increased, while in Tie1 knockout mice, the above effects were significantly weakened. Tie1 does not bind to angiopoietin directly, but co-precipitates with Tie2 to form a complex at the cell-cell junction (48). The interaction between Tie1 and Tie2 can regulate Ang-induced Tie2 phosphorylation and both Ang1 and Ang2 can increase this interaction through the ectodomain of Tie1 (47). In Tie1-deleted mouse embryos, Ang1-induced Tie2 activation decreased, leading to abnormal blood vessels and leakage (49). In the absence of pathogens, Tie1 was highly activated, and was crucial for the agonist role of Ang1 and Ang2 on Tie2 during vascular remodeling, but inflammation could lead to Tie1 inactivate through ectodomain cleavage, and reduced Ang1-Tie2 signal, and promoted the Tie2 antagonistic effect of Ang2, causing vascular destabilization (47). Although, our current model condition is hyperoxia, some studies have demonstrated that under long-term hyperoxia exposure, lung tissue could produce many inflammatory factors and oxygen radicals such as reactive oxygen species (ROS) which caused inflammatory cells aggregation on the one hand, and on the other hand, inflammatory cells could release a lot of oxygen radicals and inflammatory factors, causing a vicious cycle, leading to lung injury and eventually BPD (50). As a result, oxidative stress and inflammatory responses affected each other. For example, NF-κB is an important transcription factor that regulates inflammatory responses, studies have shown that ROS could cause rapid nuclear translocation of NF-κB, and then promote the expression of downstream pro-inflammatory factors (51). Therefore, we guessed that the changes in Ang-Tie signal were a common result of oxidative stress and inflammation. The down regulation of Ang1-Tie2 might also be related to decreased Tie1 expression.

Ang1-induced Tie2 phosphorylation stimulated the activity of several downstream signaling pathways, which stabilized the VE-cadherin at the EC-EC junction, decreased vascular permeability, and promoted cell survival and anti-apoptosis (52, 53). PI3K/Akt signaling has been demonstrated to be one of the great important downstream signaling pathways of Ang1-Tie2 pathway to play a major role in promoting ECs proliferation and survival at the early stage of angiogenesis (54, 55). The protein expression of PI3K and pAkt decreased after Tie2 inhibitor treatment, indicating that the activation of Tie2 was related to PI3K/Akt pathway transduction (56). Foxo1, can be regulated via the phosphorylation of Akt upon PI3K/Akt signaling activation, its activity is inhibited after phosphorylation (57). Strong evidence showed that Tie2 phosphorylation induced by Ang1 could phosphorylate Foxo1 via Akt, and inhibit Foxo1 transcriptional function by promoting its nuclear rejection and preventing DNA binding (15, 17). Ang1-mediated Foxo1 transcriptional function inhibition induced the expression of target genes related to vascular stability and down-regulated the expression of Ang2 and other genes related to endothelial instability, apoptosis, metabolism, and growth control (15–18), therefore, promoting vascular permeability. It has also been found that in acute inflammatory conditions, in which Ang1 and pAkt levels reduced, decreased Tie2 signaling (including Tie1 deletion) alleviated the inhibition of Foxo1 activity, resulting in increased expression of Ang2 and vascular instability, which is a typical manifestation of vascular leakage (47, 58). In this research, our western blot result showed that at 7 and 14 d, hyperoxia significantly reduced the total protein level and phosphorylation state of Akt and Foxo1 in the lung tissue, suggesting that hyperoxia downregulated the activity of Akt/Foxo1 signaling, probably increasing Ang2 transcription. Wu et al. (59) first proposed the view that Foxo1 could directly regulate Twist1. In the study of prostate tumors (PCa), the increased expression of monoamine oxidase A (MAOA) promoted the production of epithelial-to-mesenchymal transition (EMT), hypoxia, and ROS, which jointly promoted the tumorigenesis, development, and metastasis of PCa. This process was related to MAOA increasing Twist1 expression by promoting Akt-dependent Foxo1 phosphorylation to activate Twist1 transcription. Therefore, we speculated that in this research, downregulation of Twist1 and upregulation of Ang2 in hyperoxia group might probably be related to the decreased Akt-Foxo1 signaling.

**Figure 9:**
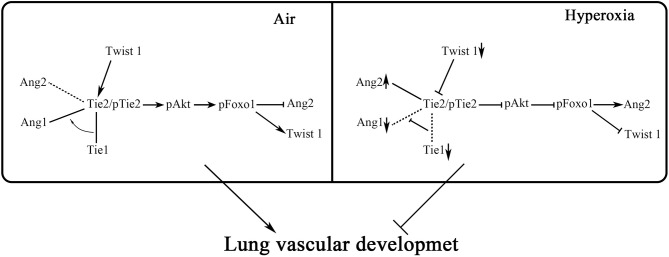
Hypothesis diagram of the regulatory mechanism of Twist1, Tie1, Tie2, Angs, and Akt/Foxo1 in endothelial cells. Twist1-Tie2-Akt/Foxo1 pathway combined to Angs signal may play an important role in hyperoxia-induced increase of pulmonary microvascular permeability. We hypothesize that hyperoxia impaired pulmonary vascular permeability may be associated with disruption of Ang1-Tie2 signaling and decreased Akt/Foxo1 pathways that increase Ang2 production, and the Twist1 and Tie1 may be involved in the regulation of this process.

In conclusion, our research showed that in HALI, the pulmonary microvascular permeability was increased, accompanied by changes in Twist1-Tie2 pathway which combined to Angs, and downregulation of Akt/Foxo1 pathway. We could speculate that Twist1-Tie2-Akt/Foxo1 pathway combined to Angs signal might play an important role in hyperoxia-induced increase of pulmonary microvascular permeability. In this study, we hypothesized that hyperoxia impaired pulmonary vascular permeability may be associated with disruption of Ang1-Tie2 signaling and decreased Akt/Foxo1 pathways that increased Ang-2 production, and the Twist1 and Tie1 may be involved in the regulation of this process (Figure 9). However, this study did not involve direct evidence supporting the direct relationship between pulmonary microvascular permeability and Twist1-Tie2-Akt/Foxo1signal activity in HALI. There needs to be *in vivo* as well as *in vitro* (using pulmonary microvascular endothelial cells) Twist1 knockdown experiments conducted to further explain the mechanistic role of Twist1 pathway in lung microvascular permeability in HALI. Our experimental data just provide further experimental evidence for the important role of the Twist1 signaling pathway on microvascular permeability in hyperoxia-induced lung injury.

## Data Availability Statement

The datasets generated for this study are available on request to the corresponding author.

## Ethics Statement

All animal procedures were reviewed and approved by the Laboratory Animal Ethics Committee of the Southwest Medical University and conducted according to the AAALAC and the IACUC guidelines.

## Author Contributions

All authors fulfill the journal's author requirements. YR conceived, designed, and performed the main experiments. YR, LK, RZ, FW, and XZ had a substantial contribution in acquisition of the main data. YR had a substantial contribution in interpretation and analysis of the data. YR, WD, and XL drafted and revised the article. All authors approved the final version before submission.

## Conflict of Interest

The authors declare that the research was conducted in the absence of any commercial or financial relationships that could be construed as a potential conflict of interest.

## Abbreviations

BPD: bronchopulmonary dysplasia
HALI: hyperoxia-induced acute lung injury
Tie2: tyrosine kinase receptor 2
HE: hematoxylin and eosin staining
MLI: mean linear intercept
IF: immunofluorescence
EBD: Evans blue dye
PDGFB: platelet-derived growth factor B
VEGFR2: vascular endothelial growth factor receptor 2
L-HMVE: lung human microvascular endothelial
ROS: reactive oxygen species
MAOA: monoamine oxidase A
EMT: epithelial-to-mesenchymal transition.
